# Marginal Likelihood Estimate Comparisons to Obtain Optimal Species Delimitations in *Silene* sect. *Cryptoneurae* (Caryophyllaceae)

**DOI:** 10.1371/journal.pone.0106990

**Published:** 2014-09-12

**Authors:** Zeynep Aydin, Thomas Marcussen, Alaattin Selcuk Ertekin, Bengt Oxelman

**Affiliations:** 1 Department of Biological and Environmental Sciences, University of Gothenburg, Gothenburg, Sweden; 2 Department of Biology, Faculty of Sciences, University of Dicle, Diyarbakir, Turkey; University of California, San Diego, United States of America

## Abstract

Coalescent-based inference of phylogenetic relationships among species takes into account gene tree incongruence due to incomplete lineage sorting, but for such methods to make sense species have to be correctly delimited. Because alternative assignments of individuals to species result in different parametric models, model selection methods can be applied to optimise model of species classification. In a Bayesian framework, Bayes factors (BF), based on marginal likelihood estimates, can be used to test a range of possible classifications for the group under study. Here, we explore BF and the Akaike Information Criterion (AIC) to discriminate between different species classifications in the flowering plant lineage *Silene* sect. *Cryptoneurae* (Caryophyllaceae). We estimated marginal likelihoods for different species classification models via the Path Sampling (PS), Stepping Stone sampling (SS), and Harmonic Mean Estimator (HME) methods implemented in BEAST. To select among alternative species classification models a posterior simulation-based analog of the AIC through Markov chain Monte Carlo analysis (AICM) was also performed. The results are compared to outcomes from the software BP&P. Our results agree with another recent study that marginal likelihood estimates from PS and SS methods are useful for comparing different species classifications, and strongly support the recognition of the newly described species *S. ertekinii*.

## Introduction

Species is often regarded as a fundamental biological unit. A major endeavor of the field of systematics is the discovery of biological diversity and its assignment to the species category [1]–[3]. However, the lack of a unique definition complicates the recognition of particular species and therefore sometimes causes confusion among users of taxonomy including evolutionary biologists, population geneticists, and conservation biologists [4]. Species recognition is especially challenging among closely related taxa with little differentiation due to recent divergence [5], [6]. In most cases, species have been recognized primarily on morphological traits. However, as such traits may be under control of many different factors (e.g., genetic, epigenetic, environmental), the use of morphological data alone may underestimate the “real” number of species [7]–[10], and it is hard to devise an explicit, testable model based on such data alone.

DNA sequence data is potentially useful for delimiting species objectively [11]–[13]. Given multilocus data, species phylogenies can be estimated accurately [14], [15] in the presence of incomplete lineage sorting, and several recent [12], [16]–[20] studies have attempted to infer species limits based on such models in various taxonomic groups.

Coalescent theory [21] offers an appropriate approach to estimate species phylogenies from a collection of gene trees by taking incomplete lineage sorting into account [22]–[25]. The coalescent model was originally formulated to analyse the genes of a single species [26], but it was generalized to multiple species by applying the constraint that divergence between two species can not be older than the most recent coalescence time of shared alleles [22]. The multispecies coalescent (MSC) model provides a powerful probabilistic framework to explore the shape and patterns of species trees by taking into account demographic parameters that formed the ancestral history of populations. The model has become a major focus in phylogenetics and speciation research and is implemented by a number of promising Maximum Likelihood, Bayesian, and summary statistics based methods. See [27], [28] for reviews.

Although they use the same underlying MSC model, current Maximum likelihood methods such as STEM [24] and spedeSTEM [29] evaluate species trees by using fixed individual gene trees calculated from any standard phylogenetic method as input data. Bayesian methods including BEST [30], BP&P [13], and *BEAST [15] generate posterior probabilities for all parameters directly from the input sequence data by specifying prior distributions for the parameters. BEST and *BEAST utilize the Markov Chain Monte Carlo (MCMC) algorithm to jointly estimate a species tree topology and its underlying model parameters in addition to the gene trees of multiple loci sequenced from multiple individuals. Thus, under these methods, each estimated species tree topology is conditioned on the assignment of alleles to species.

Although the implementation of MCMC enables posterior distributions conditioned on particular sets of parameters to be estimated, testing of alternative models is not as straightforward. For example, changing the number of species given the same set of sequences will change the number of branch parameters in the species tree. Therefore, the problem of species delimitation can be viewed as a problem of model selection. One way of comparing different models is to use Bayes Factors (BF), which is the ratio of the marginal likelihood of one model to the marginal likelihood of a competing model, where the marginal likelihood measures the average fit of a model to the data. Recently, one popular method for marginal likelihood estimation that has been widely used in phylogenetics, the Harmonic Mean Estimator (HME) [31], was reported to produce biased estimates and therefore failing to yield a reliable result [33], [34], [35]. On the other hand, two other relatively newly developed methods, Path Sampling (PS) [32], [33] and Stepping Stone sampling (SS) [34], have been shown [35], [36] to generate highly accurate results for the assessment of choices of molecular clock [37], [38] and demographic models [39].

Grummer et al. [20] showed that besides increasing the accuracy of phylogenetic inference through model selection, marginal likelihood estimates also should allow to choose among a set of species delimitation hypotheses where each hypothesis is a competing model of assignment of sequences to a certain set of species. Since the marginal likelihood provides the fit of the model to the data, the species delimitation model with the highest marginal likelihood will be the one fitting the data best. This approach has certain advantages over the approach using reversible model jump MCMC (rjMCMC) to simultaneously infer marginal probabilities for nested MSC species classification models, given a fixed guide tree of “minimal” species proposed by Yang and Rannala [13]. One such advantage is that Marginal likelihood comparison does not require alternative species delimitation models to be nested. Another advantage is that the user is not dependent on a predefined and possibly inaccurate guide tree.

### Species concept under the MSC

Integration of powerful statistical methods with the MSC model not only provides replicable results but also gives a conceptual perspective to species recognition that enables objective testing of particular hypotheses of species delimitation. As implemented in the MSC model, species are independently evolving population lineages. This satisfies the criteria of several species concepts that all are covered by the general lineage concept [4]. Species in this model are defined by abrupt speciation and no genetic exchange after the speciation event, similar to the biological species concept [40], but in retrospect. Thus, in the multispecies coalescent model, species constitute the branches of the species tree and are in principle testable through the statistical nature of the model. Current implementations of the model may be unrealistically simple, but it does provide an objective basis. We believe that with advances in computational techniques that may enable more realistic parameterization of the MSC model, taxonomic discovery, resolution and consistency in the results will increase and ultimately provide an objective ground for taxonomic stability for any field which relies on accurate measures of biodiversity.

*Silene* L. (Caryophyllaceae) is a large genus of flowering plants and is attractive as a model system for studies of among other things evolution of sex chromosomes, breeding system, pollination, and aberrant evolution of mitochondrial genes [41]–[43]. However, the taxonomy in the genus has been highly controversial and almost none of the 44 sections in the most widely cited global revision [44] are congruent with phylogenetic relationships observed from molecular data e.g., [45]–[47]. A dynamically updated revised classification is kept at www.sileneae.info [48].

*Silene insularis* Barbey, *S. salamandra* Pamp., *S. cryptoneura* Stapf, and *S. ertekinii* Aydin and Oxelman are morphologically highly similar [49] and belong in *Silene* sect. *Cryptoneurae* Aydin and Oxelman. The current species delimitations in the group are mainly based on floral characteristics (e.g., carpophore length, calyx shape, anther size) and geographical distribution. Although DNA sequence data have indicated a close relationship among the species [45], [50], their phylogeny and delimitation have never been investigated extensively.

*Silene insularis*, endemic to the SE Aegaean island of Karpathos, is recognized by being smaller in some floral characteristics (e.g., carpophore length, petal size, stamen length, anther size) presumably related to autogamy, compared to the other taxa. *S. salamandra*, endemic to the island of Rhodes, and has a shorter carpophore (2–3 mm) than the SW Anatolian taxa *S. cryptoneura* and *S. ertekinii* [49], [51], [52]. The latter two occupy virgin habitats of the medium altitude zone of the Burdur, Lycia, and the Western Antalya provinces [53] and have been treated as a single species until Aydin et al. [52] separated the populations occurring to the east of the Bey mountains as a separate taxonomic species, *S. ertekinii*.

In this paper, we aim to optimize species delimitation in *Silene* sect. *Cryptoneurae* under the MSC model with special focus on the application of three marginal likelihood estimation methods HME, PS, SS, in addition to the a posterior simulation-based analogue of AIC through MCMC (AICM) [54].

## Materials and Methods

### Plant material

**Figure 1:**
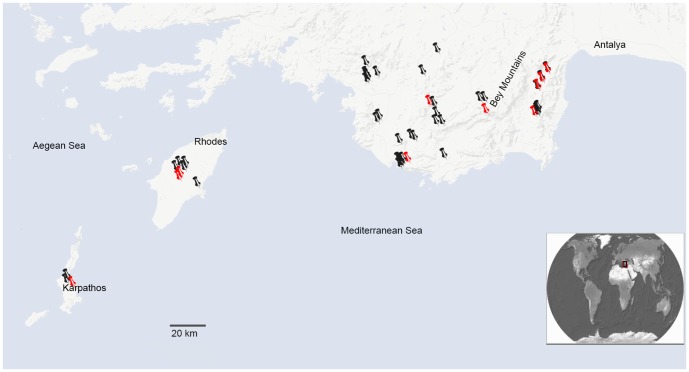
Map of Southwest Anatolia and Aegean Islands of Rhodes and Karpathos showing the known geographic distributions of *Silene* sect. *Cryptoneurae*. Icons are corresponded to the locations of specimens examined for this study. Red icons are corresponded to the locations of the specimens used in our molecular analysis.

The plant material used for DNA extraction is listed in the Electronic supplementary material (Table A in File S1). Geographic locations of these samples are shown in Figure 1. For simplicity, we have used the acronyms E (*S. ertekinii*), W (*S. cryptoneura*), S (*S. salamandra*), I (*S. insularis*) as names for these units, which we consider as “minimal species” for the purpose of this paper.

### Molecular Methods

DNA was extracted from dried plant material with the E.Z.N.A SP plant mini Kit (Omegabiotek) following instructions from the manufacturer. In cases where extraction difficulties were encountered, a modified CTAB protocol [45] was used. In these extractions, total DNA was purified using the Glass Milk kit (Q Bio-Gene, Solon, Ohio, U.S.A.) following the manufacturer's guidelines.

From each sample, sequence data were generated for six loci: one chloroplast (*rps16*) and five potentially unlinked low copy nuclear regions (*NRPA2, NRPB2, EST04, EST14*, *EST24*). For *NRPA2*, *NRPB2*, we amplified introns using the primers developed by [55] while *EST04, EST14, EST24* are newly developed regions from EST libraries of *Silene uralensis* (Rupr.) Bocquet and *S. schafta* J.G.Gmel. ex Hohen [56]. See http://www.sileneae.info/annas/GEM_EST.html for gene annotations. Primer sequences are listed in Electronic supplementary material Table B in File S1.

PCR amplifications were performed with the high fidelity DNA polymerase kits Phusion (Finnzymes) for *NRPA2* and *NRPB2* regions and Platinum (Invitrogene) for *rps16* and the EST loci, according to the manufacturer's instructions.

Amplified products were purified with Multiscreen PCR plates in a vacuum manifold (Millipore) and sent to Macrogen Inc. in Seoul, South Korea for Sanger sequencing.

*NRPA2* and *NRPB2* sequences were visually edited via Staden v.1.6.0 [57] in combination with Phred v.0.020425.c [58] and Phrap (www.phrap.org) and EST regions were edited in Geneious Pro 5.4.6 [59]. Double peaks in the chromatograms were interpreted as base polymorphisms where the lower peak was at least half of the height of the higher peak and visible in both sequence directions. PCR products with sequences with more than one polymorphic site were re-sequenced with allele-specific primers [60], or *in vivo* cloned either with Qiagen (Sollentuna, Sweden http://www.qiagen.com) or the TOPO TA (http://www.invitrogen.com) cloning kit for sequencing, to separate the sequence copies (“alleles”).

### Multiple Alignments

Multiple sequence alignment was performed with MUSCLE as implemented in Geneious version 5.4.6 under default settings. Alignments were then optimized manually to make sure that indels having identical length and position were consistently aligned. Indel characters were coded via SeqState [61] by selecting the simple indel-coding [62] option as implemented in the software. Each alignment file was checked for number of segregating sites, parsimony informativeness, and consistency index in PAUP version 4.0b10 [63]. Information on alignment files are summarized in the Electronic supplementary material Table C in File S1.

### Phylogenetic analysis

Nucleotide substitution model for each locus was selected informed by the AICc (Akaike's information criterion) criterion in Modeltest version 3.8 [64] as implemented in PAUP* version 4.0b10 [63]. In order to detect possible recombination within loci, each nuclear data set was analysed with Dual Brothers [65] implemented in Geneious version 5.4.6, and GARD (Genetic Algorithm for Recombination Detection [66]), online available at www.datamonkey.org/GARD.

Bayesian gene phylogenies were estimated using BEAST v1.7.5 [67]. Indel data were coded as binary characters and run under the time reversible binary substitution model. For the substitution model, priors given by BEAUti v. 1.6.2 were accepted, and same priors were used for the models that had to be edited by hand in the xml files. Each data set was run separately with a strict and an uncorrelated lognormal clock model. The lognormal model had a prior mean 1.0 and standard deviation 1.25. Because gene trees within MSC species are expected to follow a coalescent model, whereas branches between species are expected to follow a birth/death model, each data set was also checked for the appropriateness of the Yule process or the coalescent constant size as the tree prior. In the case of coalescent constant size, a gamma prior with shape = 2.0 and scale = 0.002 was set on the population size, otherwise default priors were accepted.

For each gene, four xml files (see supplementary File S2) were generated and subsequently run for 15 million generations. Due to convergence problems, additional runs with 50 million generations were performed on the *EST24* data set. The first 10% of the generations were discarded as burn-in. For *EST24* every 5000th and for the other loci every 1000th iteration from the remaining chain were sampled to estimate the posterior distributions. Convergence and mixing of each run were assessed with Tracer v1.5.0 (http://tree.bio.ed.ac.uk/software/tracer/) [68]and followed by processing of the tree samples with TreeAnnotator v1.7.5, using the mean node heights to construct the maximum clade credibility tree with a minimum clade credibility value of 0.5. In general, the Effective Sample Sizes (ESSs) for each sample as reported by Tracer were well above 800. Maximum clade credibility trees were edited in FigTree v1.4.0 (http://tree.bio.ed.ac.uk/software/figtree/).

To be able to set the best clock model and the best tree prior on each gene for species tree estimation analysis, we calculated marginal likelihoods via the Path Sampling (PS) and Stepping Stone (SS) sampling methods [35], [36]. Marginal likelihood was estimated from 100 path steps, each run for 15 million generations. A difference of more than 3 log likelihood units (considered as “strong evidence against competing model” by [69]) was used as threshold for accepting a more parameter-rich model.

We used *BEAST [15] for the estimation of species phylogenies. To investigate different possible species delimitations and relationships among four lineages identified as species by [52], we defined nine different possible species delimitation models (see Table 1) to compare via *BEAST as implemented in BEAST 1.7.4. Note however, that *BEAST requires at least two species, so the one-species classification had to be implemented using the coalescent constant model with unlinked substitution models, clocks, and trees for the genes in BEAST.

**Table 1:** Nine species classification models compared for marginal likelihoods.

Model	Species delimitation	Number of included species
**1**	EWSI	(1 species)
**2**	EW+S+I	(3 species)
**3**	E+W+S+I	(4 species)
**4**	E+W+SI	(3 species)
**5**	E+WSI	(2 species)
**6**	ESI+W	(2 species)
**7**	EW+SI	(2 species)
**8**	EWI+S	(2 species)
**9**	EWS+I	(2 species)

Each model represents a possible relationship for *Silene* sect. *Cryptoneurae* with varying number of species. Abbreviations correspond to *S. ertekinii* (E), *S. cryptoneura* (W), *S. salamandra* (S), and *S. insularis* (I).

For each classification, substitution models for each locus were defined as shown in Table C in File S1. Priors were set as for the individual gene tree analyses. For all classifications except number one, gamma priors (shape = 2, scale = 0.002) was applied to the population sizes (piecewise linear and constant root) and the species tree birth rate. For classification 1, a gamma prior with shape = 5, and scale = 0.2 was set on the population size. Indel and DNA data were linked for tree and clock models and unlinked for the substitution model. Since *EST24* has the largest fraction of segregating sites, it was set as having mutation rate equal to 1.0, and rates for other genes were estimated relative to this. Ploidy level was set to 2 for the nuclear loci, and 1 for the chloroplast locus (the plants are hermaphrodites). Following the BF comparisons, a relaxed clock was set on the *NRPB2* locus and a strict clock was set on the other loci. Each classification model was run for 20 million generations with two replicates where the first 2 million iterations was discarded as burn-in and every 1000th generation from the remaining chain was logged. Tracer v1.5.0 was used for evaluation of convergence and mixing. ESSs for priors, posteriors, likelihoods, etc. were always considerably higher than 300.

We estimated marginal likelihoods for each model of classification using the HME, PS, and SS methods. PS and SS analyses were conducted with 80 path steps, each run for 500,000 generations. In addition to these we calculated AICM scores as described in [35]. Tree files from independent runs were combined with LogCombiner v1.7.1 before summarized with TreeAnnotator v1.7.1 and displayed with Figtree v1.4.0.

We also used the program BP&P [13], [70] version 2.2 for comparisons of the results from the marginal likelihood estimates of the different species delimitations. We checked for the posterior distributions for speciation events along of all 15 possible rooted guide trees for the four minimal lineages. We used a gamma prior G(2, 2000) on the population size parameters. The age of the root in the species tree (τ_0_) was assigned a gamma prior G(2, 2000), while the other divergence times were assigned a Dirichlet prior ([13]: equation 2). Ploidy level was set to 2 for the nuclear loci, and 1 for the chloroplast locus. We did one set of runs with substitution rates equal among loci, and one with variable rates according to a Dirichlet distribution with alpha = 5.

We ran three separate runs for each possible guide tree topology for 20 million generations. The first 2 million of iterations were discarded as burn in, with sampling frequency of every 200 from the remaining chain. To enhance comparability with BP&P (see below), we did additional *BEAST runs with the JC69 substitution model for each of the loci (10 replicates per delimitation model), as it is the only model implemented in BP&P.

All models were run without data to check for spurious prior distribution interactions. All MCMC runs were given different pseudorandom numbers to get initial starting parameter values.

## Results

There was no statistically significant evidence of recombination for any of the loci. GARD detected single breakpoints for *EST24*, *EST14*, and *EST04*, respectively, but none of them was found statistically significant (p>0.1). Results obtained from GARD were consistent with the results from DualBrothers.

The Bayesian estimation of single gene phylogenies showed that the gene trees generated under the coalescent constant prior produced much higher Bayes factor (12<BF<49) than those generated under Yule model across all the loci. Only in the *NRPA2* locus a relaxed clock was favored over a strict clock with a difference of more than 3 units. The Bayesian gene tree phylogenies consistently placed the sequences of E on a separate clade. For *NRPB2*, *EST14*, and *rps16* the W samples were sister to the clade including S and I, but in the *NRPA2, EST04*, and *EST24* loci they were intermingled with I in a clade, sister to S (see electronic supplementary material S3).

**Figure 2:**
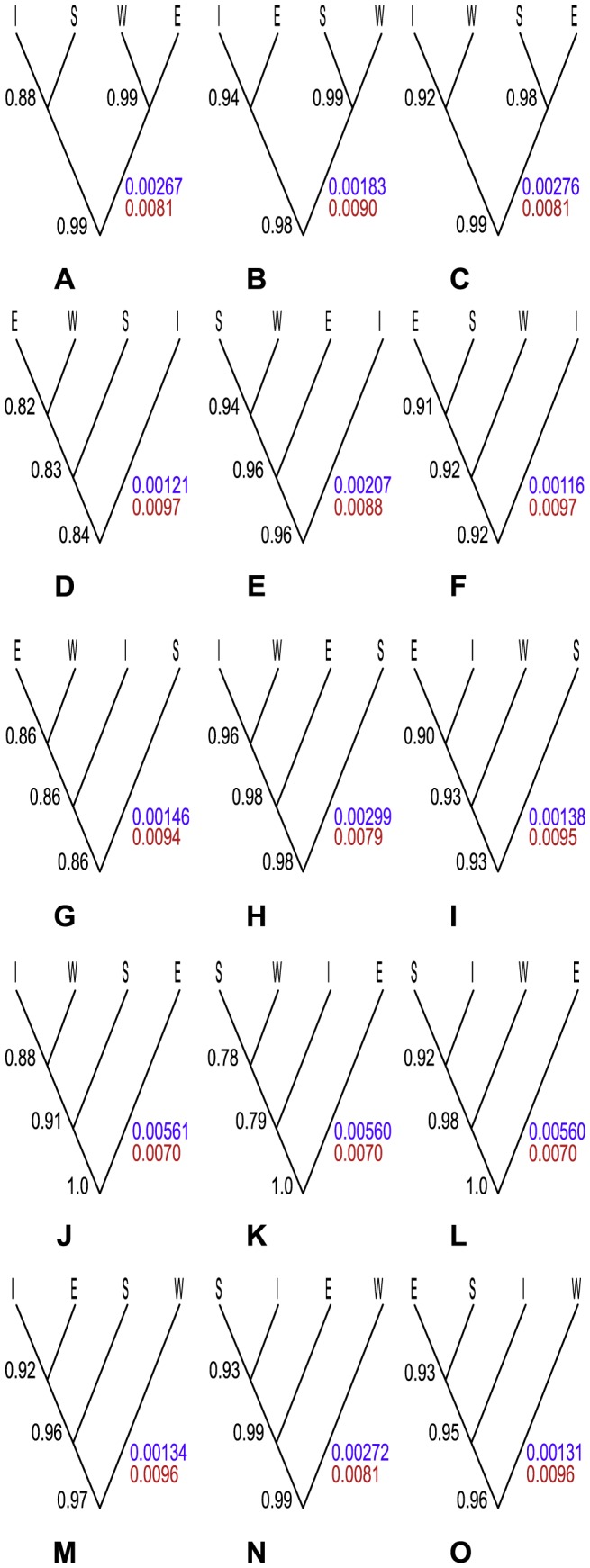
Results of 15 guide trees evaluated with BP&P. A–O. Numbers to the left of the nodes are the means of posterior probability values for speciation in that particular node observed from three replicate analyses. Colorful numbers to the right of each tree are mean values from the three separate runs for the root height (purple) and effective population size (red) of that particular tree. Tip abbreviations correspond to *S. ertekinii* (E), *S. cryptoneura* (W), *S. salamandra* (S), and *S. insularis* (I).

The BP&P analysis strongly (>0.99 posterior probability (PP)) supported speciation at the root of the guide trees into the E lineage and the rest, no matter how the other three lineages were related (Figure 2J–L). The root heights were around three times higher for this class of guide trees than for the other, and the population sizes were slightly smaller. Divergence of E followed by the W lineage as the second split was also strongly supported (0.98 PP), but when W swapped place with either I or S, the support decreased (Figure 2J–K). Setting S and I as the basal splits resulted in rather low support for all the nodes in the guide tree (Figure 2D, G). All other guide trees revealed relatively high support (≥ 0.90 PP) for speciations at all nodes despite the fact that our analyses without data gave posterior probabilities for these nodes as expected from the prior. Setting the locus rate as fixed slightly decreased the posterior probabilities for most of the nodes in the guide trees, but this difference was not significant enough to be conclusive.

**Figure 3:**
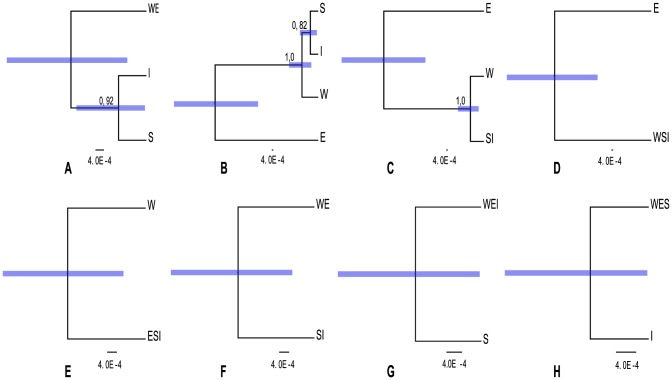
8 Species delimitation models estimated with *BEAST. A–H shows delimitation models in the order 2, 3, 4, 5, 6, 7, 8, 9 (Table 1). Tip label abbreviations are corresponding to *S. ertekinii* (E), *S. cryptoneura* (W), *S. salamandra* (S), and *S. insularis* (I). The bars on the nodes show the 95% Highest Posterior Density (HPD) of the height. Numerical values above nodes are posterior probabilities values for that particular node. Scale bar is fixed and displayed in units of substitutions per site.

The *BEAST estimation of species relationships according to each of the nine classification schemes (Table 1) are summarized in Figure 3. For model 2 (Figure 3A), when the E and W sequences were constrained to belong the same species, the branch leading to the island lineages S and I got moderate (0.92 PP) support. In model 4 (Figure 3C), where S and I were constrained to be the same species but the E and W sequences assigned to belong to different species, the branch that separates the W and island lineages from the E lineage got very strong (1.0 PP) support.

**Figure 4:**
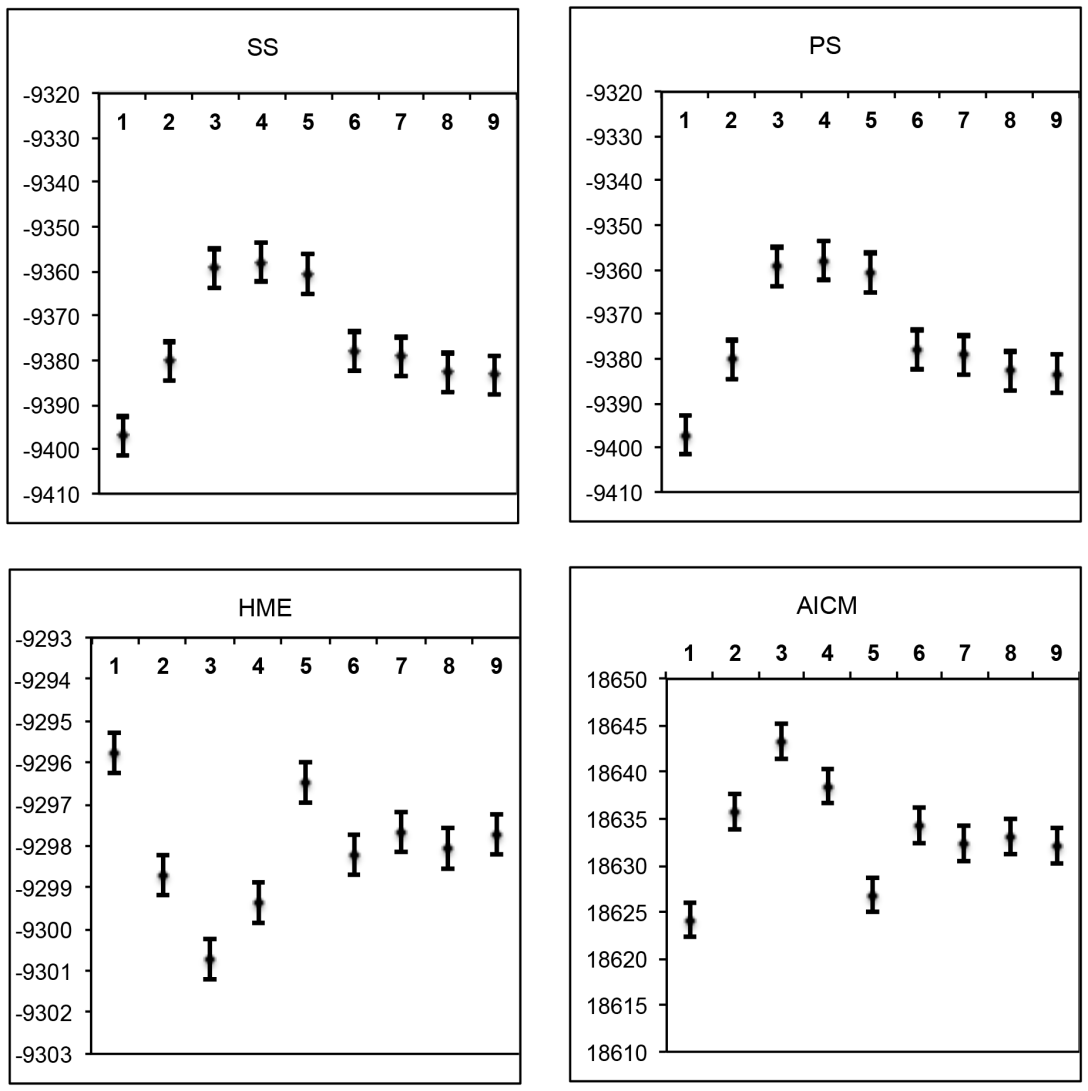
Means and 95% confidence intervals of marginal likelihood estimates and AICM values estimated from 10 replicate analyses for each of the classification model (1–9). Marginal likelihood were estimated via Path Sampling (PS), Stepping Stone (SS), and Harmonic Mean (HME) methods. AICM (a posterior simulation-based analogue of AIC through MCMC) values were obtained through AICM test.

We found significant differences among the Marginal likelihoods calculated by different methods as well as the AICM, although all the methods revealed consistent results across ten replicates of each classification model (Figure 4). HME displayed results that were contradictory to PS and SS, had less variance and much higher means. This is in accordance with previous studies (e.g., [33], [34], [35]), which have shown that HME overestimates the marginal likelihood. PS and SS had very similar results, and produced the highest marginal likelihoods for the classification models 3, 4, and 5 (Figure 3B–D). The AICM method gave results similar to HME (note that the scale is inverted for AIC).

The *BEAST analysis of the nine classifications under the settings specified according to the best clock and substitution models for the gene trees, increased the posterior probabilities but did not cause any change on the tree topology comparing to topologies those observed from the analysis run under the JC69 model for all the loci. However, although there was a significant increase in the marginal likelihoods estimated by PS, SS, HME and a decrease of AICM values, the relative pattern comparing the different classifications was the same (see electronic supplementary material Table D in File S1).

## Discussion

### *BEAST and BP&P

Bayesian methods have been advocated as being more objective compared to traditional taxonomic applications of species delimitation [29] and they are being increasingly popular. Several recent studies [16]–[20], [71] have applied a number of different methods including these to infer species limits in various taxonomic groups. It has been argued that species limits should be evaluated by using a wide range of available methods and decisions should be made by trusting on observable congruence across methods, as this will give robustness to a particular species classification [72]. However, the results from each method are only valid under its own assumptions. The use of many different methods raise the difficulty of interpreting results, especially when there is large incongruence among these. Therefore, if an estimate of a species phylogeny is the goal, species should be delimited to maximize the fit to the particular phylogeny model. Marginal likelihoods estimates for alternative species delimitation models under the MSC can be compared to identify the optimal species classification for the group under study.

Currently available species delimitation methods fall into two general classes [29], [72]. Validation approaches (i.e., BP&P, SpedeSTEM, Bayes Factor comparisons) require that samples (alleles) be assigned to putative species prior to analysis, while discovery approaches (i.e., Structurama [73]; Brownie [6]) do not require this [72]. If existing evidence can not provide a clear delineation of lineages, the use of discovery methods may be necessary. However, not all discovery methods take the MSC model into account. In principle, the use of BF comparisons also prevents users being dependent on *a priori* definitions of lineages, but the number of possible classifications [6] may restrict the number of models that can be tested in practice.

### Bayes Factors

As in [20], we employed BF as they are used in formal model selection (e.g., [35]), to compare different classification models implemented in *BEAST. Marginal-likelihood scores estimated for each species delimitation can vary depending on the estimator used to calculate them. The SS and PS methods gave strong support for the recognition of the E samples as a distinct species (classifications 3, 4, and 5, see figure 3). BF calculated via HME contradicted the results of the PS and SS methods. The AICM results reminded of those from HME but had higher variance. Our results seem to be in agreement with those of [20], [35] that one should use the PS and SS methods, and avoid HME and AICM. As stated by [36], it is important that proper priors (integrating to 1) are used, otherwise the marginal likelihood estimates from PS/SS can be affected by the priors. Baele et al. [74] showed that the accuracy of BF increases if one uses an SS approach to create a path between the two competing models, compared to marginal likelihood estimation of individual models, but at a significant extra cost in terms of computational demands. The results of [20] show that the approach used by us is valid at least in some situations, but more studies on species delimitation would be beneficial.

### Guide tree

Arrangement of the guide tree has critical importance for BP&P outcomes [12], [13], [75]. When alleles can be assigned to putative species unambiguously, applying a species tree estimation method can serve as selection procedure for choosing the guide tree. However, this also requires the guide tree to be estimated “correctly”, which may be hard because of poor information content of the gene and/or gene flow among terminals (the “minimal species”). The BF method does not rely on a fixed tree topology and alternative delimitation models do not have to be nested. A potential problem in our comparisons is that the *BEAST model is implemented only for two or more species [15], so the comparison with the one-species classification may be affected by other model differences. Grummer et al. [20] used an outgroup species to overcome this problem. In our case, the genetic distance to any other species are large (e.g., [45], [50]), so other problems pertaining to difficulties in reconstructing clocklike trees with long branches may be introduced if such an outgroup is included.

Evaluation of all fifteen possible topologies for four species as guide trees provided insights about the behavior of the BP&P method. Although correct specification of the guide tree is essential for the method, we conclude that the results might in some cases be robust to misspecifications. When the data are informative enough, like for the E and W lineages in the study, the method generated relatively good support for divergent lineages despite variable positions of them across the guide tree. A split between the E and W lineages, unless both of the island lineages of the group are specified as the oldest splits (Figure 2D, G), received good support (≥ 0.90) in all the guide trees. One possible explanation to this phenomenon, suggested by [12], [20], may be that divergent lineages in the tree which are not sister lineages will always be supported as different species. If one assumes that the island lineages are the two youngest lineages of the group, and put them as the deepest split in the tree, this will require their alleles to coalesce earlier than the first speciation node, which seems unlikely for those near-identical alleles in the group. The posteriors for nested speciation events will always be conditioned on the more ancient events, as speciation events can never occur in trees where the preceding speciations do not occur. Therefore, if one “normalizes” the posterior probabilities against the one obtained at deeper levels, support for speciation events can be detected, even if the guide tree is misspecified.

### Species delimitations in *sect. Cryptoneurae*

The results from our study show some support (e.g., Figure 3B, C) for W being distinct from the E and island lineages, in agreement of the current taxonomic recognition of *S. cryptoneura*. The poor resolution for the position of the island lineages may be due to poor sampling, which makes it difficult to clearly resolve the phylogenetic position of these three species in the group. In particular, *S. cryptoneura* and *S. salamandra* are very similar morphologically, whereas *S. insularis* is easily recognized on its smaller floral parts presumably associated with self-pollination. Despite the similar morphology and habitat requirements, the observed genetic differentiation between *S. cryptoneura* and *S. ertekinii* suggests that the Bey Mountain range has acted as a geographic barrier against gene flow or hybridization [52]. In agreement with the current taxonomic recognition of *S. salamandra* and *S. insularis* [47], the island lineages S and I turned out as sister lineages sharing a common ancestor with *S. cryptoneura*, although the support for this relationship was poor.

In conclusion, our study provides support for the recognition of the newly erected species *S. ertekinii*. It also concurs with [20] in that marginal likelihood estimation of different species delimitation models may provide an important source of information to taxonomy, and be a valuable validation approach for choosing among species classification when attempting to reconstruct phylogenies under the MSC model.

## Supporting Information

File S1 **Table A–D**.
(DOCX)

File S2 **BEAST XML files**.
(ZIP)

File S3 **Maximum clade credibility gene trees with posteriors clades, in NEXUS format.**
(ZIP)
