# Correction: Marginal Likelihood Estimate Comparisons to Obtain Optimal Species Delimitations in *Silene* sect. *Cryptoneurae* (Caryophyllaceae)

**DOI:** 10.1371/journal.pone.0116266

**Published:** 2014-12-17

**Authors:** The PLOS ONE Staff

There are several errors in this article. Figure 4 is incorrect. The corrected Figure 4 can be viewed here.

**Figure 4:**
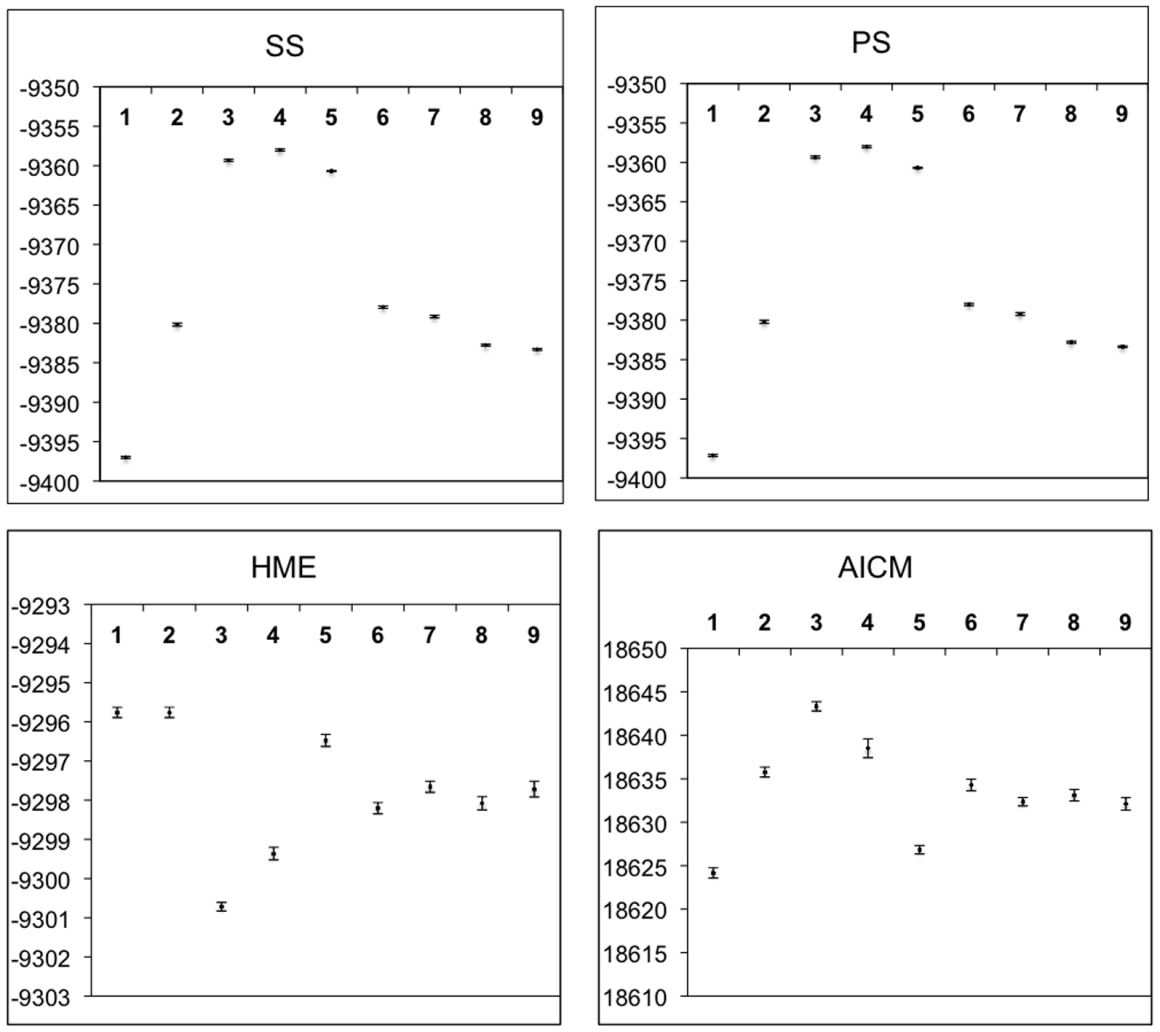
Means and 95% confidence intervals of marginal likelihood estimates and AICM values estimated from 10 replicate analyses for each of the classification model (1–9). Marginal likelihood were estimated via Path Sampling (PS), Stepping Stone (SS), and Harmonic Mean (HME) methods. AICM (a posterior simulation-based analogue of AIC through MCMC) values were obtained through AICM test.

In the second to last paragraph of the Results section, the second sentence is incorrect. The correct sentence is: “HME displayed results that were contradictory to PS and SS, and had much higher means.”

In SI Table 1C, the value for the length column of Est 14 is incorrect. The corrected value is 1547.
