# Supplementary material for: Marginal Likelihood Estimate Comparisons to Obtain Optimal Species Delimitations in Silene sect. Cryptoneurae (Caryophyllaceae)
Source: PLoS One. 2014 Sep 12;9(9):e106990. doi: 10.1371/journal.pone.0106990 (PMC4162574; doi:10.1371/journal.pone.0106990)
Supplement: File S1 — Table A–D. (DOCX) [file pone.0106990.s001.docx]

| Specimen name and ID | Geographic location | Collection year and herbarium | Collector and number | Gen Bank accession # |
| --- | --- | --- | --- | --- |
| *Silene cryptoneura* 1115 | Antalya, Finike - Elmalı 38 km.,36.52 / 30.02 | 1988 - GB | Bengt Oxelman 1628 |  |
| *Silene salamandra 2410* | Rhodes, 4km SW of Alaerma, 36.12 / 27.90 | 1993 -GB | Bengt Oxelman 2205 |  |
| *Silene ertekinii* 1177 | Antalya, Antalya-Altınyaka, 36.68 / 30.43 | 1988 -GB | Bengt Oxelman 1691 |  |
| *Silene ertekinii 15352* | Antalya, Altınyaka  36.65 / 30.40 | 2009 - GB | Zeynep Aydin 28 |  |
| *Silene cryptoneura 14858* | Antalya, W Gömbe  36.56 / 29.62 | 2009 - GB | Bengt Oxelman 2513 |  |
| *Silene cryptoneura 14849* | Antalya, Sarıbelen to Gökçeören,36.25/29.48 | 2009 - GB | Bengt Oxelman 2504 |  |
| *Silene ertekinii 15351* | Antalya, Kemer  36.51 / 30.42 | 2009 - GB | Zeynep Aydin 31 |  |
| *Silene salamandra 15345* | Rhodes, 36.13 / 27.91 | 2008 - GB | Bengt Oxelman 2541 |  |
| *Silene insularis 15372* | Karpathos,  35.57/ 27.17 | 1984 - UPS | Raus 9700 |  |
| *Silene ertekinii 15178* | Antalya, Altınyaka  36.74 /30.45 | 2009 - GB | Zeynep Aydin 36 |  |

**Table** A. Information on vouchers used in multilocus species delimitation analyses. The number at the end of the species name corresponds to specimen ID in the BoxTax database (www.sileneae.info). Locations are approximate and given with decimal GPS coordinates (N/E). Herbarium acronyms are according to Thiers (2013).

| Primer’s name | Primer’s sequence 5’ to 3’ |
| --- | --- |
| RPA2FP | GCCGTTTTCWGAGATAACTGGGATGCGT |
| RPA2RP | GRTAATAAACAGGYCCAATAAAGATCTC |
| RPA2FS | CATGCRTTTCCTTCTAGRATGAC |
| RPA2RS | GTTAAMTCGGTRCCATAAACTC |
| f7327 | CCATCYCGTATGACAATCGGYCAGCTT |
| r7586r | CCCMGTGTGACCATTGTACATTGTCT |
| Sil_EST24seq_F | AGCAAATGGGAGAAGATAAC |
| Sil_EST24seq_R | AATTCCTTGTTGCGAATGT |
| silene-est4-F | CTGCTGTTGGACAGGATTGTG |
| silene-est4-R | CATCTCCACCAGTCTCAACACC |
| Sil_EST14seq_F | GCCTCCTATTATCCTTGCAT |
| Sil_EST14seq_R | GCAACATAGACACCAGCAGT |
| rpsF | GTGGTAGAAAGCAACGTGCGACTT |
| rpsR2R | TCGGGATCGAACATCAATTGCAAC |
| rpsF2a | CTTGAAGGACATGATCTGTTGTGGA |
| rpsR3R | CGATAGACGGCTCATTGGGATA |

**Table** B. Information on the primers used in the study

| Locus | Length (bp) | S | PI | Indels | Consistency index | Substitution Model | | Bayes factors  (Strict Clock) | | Bayes factors  (Relax Clock) |
| --- | --- | --- | --- | --- | --- | --- | --- | --- | --- | --- |
| *RPA2* | 877 | 53 | 21 | 15 | 0.96 | K81uf | -1599.710533 | | -1590.307850 | |
| *RPB2* | 736 | 47 | 31 | 12 | 1.00 | K81uf | -1276.096758 | | -1274.020649 | |
| *EST04* | 691 | 30 | 22 | 3 | 0.96 | K81uf | -1170.513219 | | -1170.769777 | |
| *EST24* | 657 | 80 | 27 | 3 | 0.90 | K81uf+G | -1428.747662 | | -1428.930292 | |
| *EST14* | 2163 | 50 | 24 | 12 | 1.00 | TrN | -2344.444674 | | -2341.719368 | |
| *rps16* | 895 | 26 | 7 | 10 | 1.00 | HKY | -1260.661587 | | -1260.240044 | |

**Table** C. Aligned length (base pairs), numbers of segregating sites (S), numbers of parsimony informative sites (PI), indels, Consistency index (CI), models of sequence evolution and Bayes factors (BF) for strict and relaxed molecular clock models.

|  | _1_ | _2_ | _3_ | _4_ | _5_ | _6_ | _7_ | _8_ | _9_ |
| --- | --- | --- | --- | --- | --- | --- | --- | --- | --- |
| _PS_ | _-8783.20_ | _-8773.74_ | _-8744.91_ | _-8743.47_ | _-8742.89_ | _-8766.51_ | _-8768.10_ | _-8774.16_ | _-8774.19_ |
|  | _-8785.88_ | _-8771.04_ | _-8743.79_ | _-8743.10_ | _-8742.32_ | _-8767.58_ | _-8766.76_ | _-8771.38_ | _-8772.02_ |
| _SS_ | _-8783.65_ | _-8774.13_ | _-8745.54_ | _-8744.34_ | _-8743.11_ | _-8766.93_ | _-8768.73_ | _-8775.02_ | _-8774.76_ |
|  | _-8786.01_ | _-8771.31_ | _-8744.52_ | _-8743.80_ | _-8742.94_ | _-8768.17_ | _-8767.25_ | _-8771.83_ | _-8772.77_ |
| _HME_ | _-8631.59_ | _-8636.31_ | _-8636.44_ | _-8634.10_ | _-8631.63_ | _-8634.23_ | _-8633.38_ | _-8633.82_ | _-8633.74_ |
|  | _-8632.60_ | _-8636.39_ | _-8635.53_ | _-8633.04_ | _-8631.76_ | _-8634.28_ | _-8634.65_ | _-8634.71_ | _-8632.92_ |
| _AICM_ | _17323.3_ | _17340.4_ | _17343.8_ | _17335.4_ | _17323.3_ | _17335.4_ | _17329.5_ | _17333.7_ | _17332.5_ |
|  | _17330.0_ | _17343.6_ | _17342.7_ | _17330.6_ | _17323.7_ | _17334.5_ | _17335.0_ | _17335.4_ | _17326.9_ |

**Table** D. AICM and Marginal likelihood values for 9 species delimitation models estimated from two separate analysis run under the selected substitution and clock models. Path Sampling (PS), Stepping stone Sampling (SS), Harmonic Mean (HME), posterior simulation based analogue of Akaike’s information criterion trough MCMC (AICM).
